# Transcriptomic analysis unravels the molecular response of *Lonicera japonica* leaves to chilling stress

**DOI:** 10.3389/fpls.2022.1092857

**Published:** 2022-12-22

**Authors:** Meng Zhang, Mengxin Li, Hongwei Fu, Kehao Wang, Xu Tian, Renping Qiu, Jinkun Liu, Shuai Gao, Zhuoheng Zhong, Bingxian Yang, Lin Zhang

**Affiliations:** ^1^ Key Laboratory of Plant Secondary Metabolism and Regulation of Zhejiang Province, Zhejiang Sci-Tech University, Hangzhou, China; ^2^ College of Life Sciences and Medicine, Zhejiang Sci-Tech University, Hangzhou, China; ^3^ Department of Techonology Center, Shandong Anran Nanometer Industry Development Company Limited, Weihai, China

**Keywords:** Lonicera japonica, chilling, leaves color, calcium, brassinosteroids

## Abstract

*Lonicera japonica* is not only an important resource of traditional Chinese medicine, but also has very high horticultural value. Studies have been performed on the physiological responses of *L. japonica* leaves to chilling, however, the molecular mechanism underlying the low temperature-induced leaves morphological changes remains unclear. In this study, it has been demonstrated that the ratio of pigments content including anthocyanins, chlorophylls, and carotenoids was significantly altered in response to chilling condition, resulting in the color transformation of leaves from green to purple. Transcriptomic analysis showed there were 10,329 differentially expressed genes (DEGs) co-expressed during chilling stress. DEGs were mainly mapped to secondary metabolism, cell wall, and minor carbohydrate. The upregulated genes (UGs) were mainly enriched in protein metabolism, transport, and signaling, while UGs in secondary metabolism were mainly involved in phenylpropaoids-flavonoids pathway (PFP) and carotenoids pathway (CP). Protein-protein interaction analysis illustrated that 21 interacted genes including *CAX3*, *NHX2*, *ACA8*, and *ACA9* were enriched in calcium transport/potassium ion transport. BR biosynthesis pathway related genes and *BR insensitive* (BRI) were collectively induced by chilling stress. Furthermore, the expression of genes involved in anthocyanins and CPs as well as the content of chlorogenic acid (CGA) and luteoloside were increased in leaves of *L. japonica* under stress. Taken together, these results indicate that the activation of PFP and CP in leaves of *L. japonica* under chilling stress, largely attributed to the elevation of calcium homeostasis and stimulation of BR signaling, which then regulated the PFP/CP related transcription factors.

## 1 Introduction

*Lonicera japonica*, which is native to East Asia, is prized in China, Korea, and Japan for its pharmacological actions and horticultural value (Miller and Gorchov, 2004). The flowers of *L. japonica* are used in traditional Chinese medicine, while the stems and leaves are used in Japanese medicine (Shang et al., 2011). As an ornamental plant resource, *L. japonica* has more and more popular properties such as its sprawling habit, numerous sweetly white and golden flowers, and attractive evergreen foliage. Now *L. japonica* has been naturalized in Argentina, Brazil, Mexico, Australia, New Zealand and United States (Guo et al., 2023). A pharmacological study has reported that *L. japonica* is used as an herbal medicine with anti-bacterial, anti-endotoxin, anti-inflammatory, and antipyretic effects (Li et al., 2015). In the recent years, *L. japonica* has become one of the key materials in traditional Chinese medicine with antiepidemic effect (Zhou et al., 2020; Wang et al., 2021). There are many chemical components in *L. japonica* such as flavonoids, organic acids, volatile oils, iridoids, triterpenoids, and saponins. The content of CGA and luteoloside are the main indicators for the quality assessment of *L. japonica* (Wang et al., 2020). Previous studies have addressed at the point of revealing the biosynthesis and regulation mechanism of secondary metabolites during the floral development of *L. japonica* (Fang et al., 2020; Xia et al., 2021). A systemic study by the integration of transcriptome, proteome, and metabolome revealed the transduction mechanism of phenylpropanoids and terpenoids biosynthesis in the stages of flower development (Wang et al., 2019b). However, although the endogenous hormones-regulated color transition of petals has been indicated by a transcriptomic analysis (Xia et al., 2021), studies on improving its horticultural and ornamental value is very limited.

As a plant resource with dual properties of medicinal and ornamental effects, it would be a meaningful exploration to borrow environmental conditions to promote the accumulation of active components of *L. japonica* and affect the external phenotype of its leaves or flowers. Recently, a study showed that light intensity had a significant effect on the flavonoids accumulation in the flower buds of *L. japonica* (Fang et al., 2020). Cold stress is one of the major abiotic stresses which limit the growth and yield of crops worldwide (Ke et al., 2020). It includes chilling (0°C–15°C) as well as freezing (< 0°C) stress (Ding et al., 2020). Growing evidences suggest that cold stress not only causes alteration in physiological and biochemical parameters (phenotype, photosynthesis, and/or antioxidant enzyme activities) of plants (Zareei et al., 2021) but also affects their metabolic pathways (Li et al., 2018). Peng et al. (2019) found that the expression of genes related to flavonol biosynthesis as well as flavonol content were increased in T*etrastigma hemsleyanum* under chilling stress. Artemisinin biosynthetic pathway was also activated by chilling stress in *Artemisia annua* (Vashisth et al., 2018). Interestingly, the flavonoids and saponins content were more accumulated in the root of *Tetrastigma hemsleyanum* (Xiang et al., 2021) and *Panax notoginseng* (Xia et al., 2017), respectively, when the transformation occurred from Summer to Autumn. While Carpenter et al. (2014) not only demonstrated the reddening of *L. japonica* leaves, but also revealed the relationship between the photoprotective function of anthocyanin and leaves reddening. Cold stress induces plant response through activating signal pathways including mitogen-activated protein kinases (MAPKs), phytohormone, and oxidative pathway (Yuan et al., 2018). Ca^2+^ has been known to play critical role in cold stress response of plants (Cui et al., 2020). The triggered Ca^2+^ signals were relayed by Ca^2+^ and decoded into downstream signaling pathways like activation of MAPKs and the production of ROS (Reddy et al., 2011). Nevertheless, it is still not clear how Ca^2+^-mediated signaling interacts with other signaling pathways to regulate the accumulation of relevant secondary metabolites under cold stress in plants.

**Figure 1:**
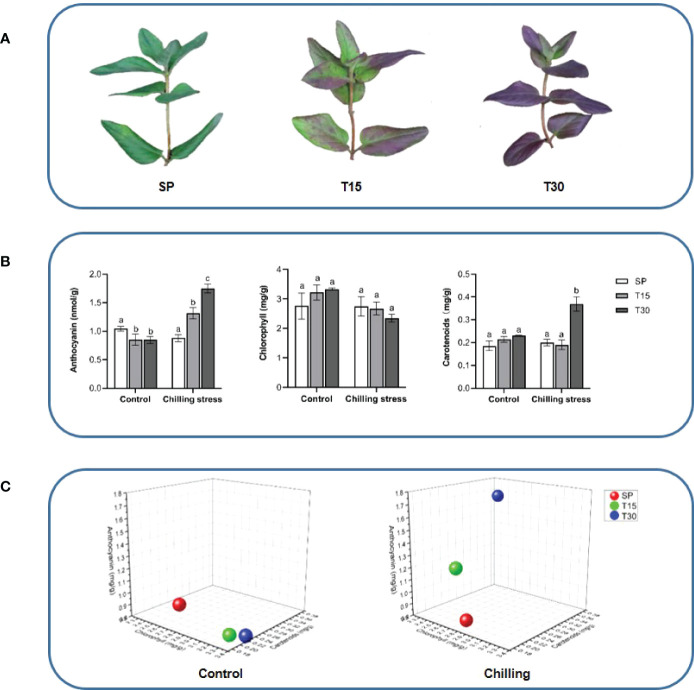
Morphological observation and pigment content of the leaves of *L. japonica.* **(A)** Phenotypic changes of leaves under chilling stress; **(B)** Pigments content analysis; **(C)** 3D coordinate map. The location of spheres in the maps represents the ratio of contents of chlorophylls, carotenoids, and anthocyanins in leaves of *L. japonica*. Means with the same letter were not significantly different among samples while different letters indicate that the change is significant according to one-way ANOVA test (p< 0.05).

In our previous experiments, an interesting phenomenon was observed that the leaves color of *L. japonica* was changed into purple when plants were transferred to a chilling environment. Preliminary experiment demonstrated that content proportion of three pigment components (anthocyanins, chlorophylls, and carotenoids) was significantly changed under the chilling stress (**Figure 1**). The accumulation of anthocyanins was suggested to be key factor for the formation of fiber color in *Gossypium hirsutum* (Gao et al., 2019; Ke et al., 2022). Here, in order to understand the inherent regulation mechanism of the phenotypic response of *L. japonica* leaves to chilling stress, transcriptome sequencing combined with bioinformatic analysis were performed. Phytochemical and qRT-PCR analyses were carried out for confirmation of transcriptomic results.

## 2 Materials and methods

### 2.1 Plant materials and chilling treatment

The seedings of *L. japonica* (Beihua No.1) were transplanted from the nursery garden in Linyi (35°18′28″N, 117°34′45″E) into potted containers and placed in a greenhouse in Zhejiang Sci-Tech University in Hangzhou (30°18′54″N, 120°21′27″E). The stem segments were cut to 5 cm in length, planted in pots with nutrient soil, and grown in an incubator (Ningbo Southeast Instrument, Zhejiang, China) with the condition of humidity of 70%, light intensity of 8,000 Lux, and temperature of 24°C in a 12 h-light/12 h-dark cycle per day. The seedlings with 8 leaves were used for chilling treatment in this study. In the treatment group, 15 seedlings were stressed with a temperature of 10°C for 30 days; in the control group, another 15 seedlings were grown at the normal temperature of 24°C for 30 days. Leaves of *L. japonica* at starting point (SP), treatment for 15 days (T15), and treatment for 30 days (T30) were collected for physiological and transcriptomic analyses. Three independent experiments were performed as biological replicates and the collected plant materials were frozen in liquid nitrogen and stored at −80°C.

### 2.2 Pigment content determination

Chlorophylls and carotenoids determination was carried out according to the method described by Luo et al. (2019) with minor modifications. Chlorophylls and carotenoids were extracted from 50 mg of leaves in 5 mL of dimethyl sulfoxide, after incubation at 65°C for 20 min (until the leaves turned white). The extraction solution was measured at 470 nm, 649 nm, and 665 nm using the UV-1800PC spectrophotometer (MAPADA, Shanghai, China). Content of chlorophylls (C_T_) and carotenoids (C_c_) were calculated using the following equations (“V” represents the final volume of the reaction and “m” represents the mass of leaves used for metabolites extraction):


$${\text{C}}_{\text{a}}=(13.95\times {\text{A}}_{665}–6.88\times {\text{A}}_{649})\times \text{V}/1000\times \text{m};$$

$${\text{C}}_{\text{b}}=(24.96\times {\text{A}}_{649}–7.32\times {\text{A}}_{665})\text{V}/1000\times \text{m};$$

$${\text{C}}_{\text{T}}={\text{C}}_{\text{a}}+{\text{C}}_{\text{b}},{\text{C}}_{\text{c}}=\left(1000\times {\text{A}}_{470}–2.05\times {\text{C}}_{\text{a}}–114.8\times {\text{C}}_{\text{b}}\right)/245\times \text{V}/1000\times \text{m}$$

Anthocyanins were extracted and determined using the method of Wang et al. (2019a) with little modification. Briefly, 50 mg of frozen leaves was grounded into powder in liquid nitrogen, sonicated with 3 ml of 0.1% methanol hydrochloride for 1 h, and then shaken overnight. After centrifugation at 2,500 g for 10 min, 1 ml of the supernatant was mixed with 1 ml of water and the mixture was further mixed with 1ml of chloroform to remove chlorophyll. The resulted solution was measured at 530 nm for anthocyanin determination.

### 2.3 Total RNA extraction, cDNA library construction, and sequencing

Total RNA was extracted from *L. japonica* leaves using an RNA extraction kit (Accurate Biotechnology, Hunan, China). The integrity of RNA was evaluated by gel-electrophoresis and the concentration and purity were determined by a NanoDrop spectrophotometer 1000 (Thermo Fisher, MA, USA). The mRNA was isolated and fragmented using the U-mRNAseq Library Prep Kit (Illumina, CA, USA). The mRNA fragments were reverse transcribed into double-stranded cDNA using Smart-RT Enzyme (Takara, Japan) and then purified with magnetic beads to repair the end of short fragments by adding a poly (A) tail and the sequencing connector. The cDNA from each group of three individuals (one per biological replicate) was pooled to build a sequencing library, which was purified using gel electrophoresis and quantitatively assayed by real-time PCR, respectively. The libraries were then sequenced by Illumina Novaseq 6000 (Illumina).

### 2.4 Gene annotation and expression analysis

Raw Data was filtered using fastp software (https://github.com/OpenGene/fastp). The adaptor, sequences with fragment length< 50 bp, reads with a certain percentage of N bases (set to 5bp by default), and low-quality bases with quality values< 20 were removed to obtain clean data. Clean data were compared to the reference genomes of *L. japonica* (Pu et al., 2020) and Arabidopsis for similarity using hisat2 (https://daehwankimlab.github.io/hisat2/). The value of fragments per kilobase of exon million fragments mapped (FPKM) was used to represent the expression level of genes. The differential expression of genes was cognized under the criterion that a significant change of gene expression between samples is identified as the fold change of FPKM value above 1 with p< 0.05.

### 2.5 Function annotation and enrichment

The function predication of genes derived from *L. japonica* was performed by transferring annotations to the Arabidopsis genome and consideration of orthologous genes. Gene functions were categorized using Mercator 4 (https://plabipd.de/portal/mercator4) (Lohse et al., 2014). Pathway mapping of identified genes was performed using MapMan software (http://gabi.rzpd.de/projects/MapMan/) (Thimm et al., 2004) and the Kyoto Encyclopedia of Genes and Genomes (KEGG) database (http://www.genome.jp/kegg/) (Kanehisa and Goto, 2000). A hierarchical clustering analysis was generated to show the fold change ratios of genes. The cluster analysis was performed using the K-Means in MeV (Multiple Experiment Viewer) (https://sourceforge.net/projects/mev-tm4/files/mev-tm4/).

### 2.6 PPI analysis

Protein-protein interactions (PPI) were generated by exporting the orthologous gene IDs of Arabidopsis to STRING (Search Tool for the Retrieval of Interacting Genes, v9.1) (https://string-db.org/). The network was displayed using cytoscape 3.9.1 (https://cytoscape.org/).

### 2.7 Phylogenetic analysis

The transcriptomic sequences of *L. japonica* and those which were obtained from NCBI were used for phylogenetic analysis. Mafft v7.464 was employed for multiple sequence alignment (Katoh and Standley, 2013) and the alignment results were used to reconstruct the phylogenetic tree by MEGA-X software (Kumar et al., 2018) with the method of Neighbor-Joining. The neighbor-joining tree was tested with 1000 bootstrap replicates.

### 2.8 Quantitative analysis of CGA and luteoloside

CGA and luteoloside were extracted from frozen-dried leaves of *L. japonica* through ultra-sonication with 2 mL methanol for 60 min. After centrifugation at 12,000 g for 10 min, the supernatant was aspirated with a syringe and passed through 0.22 μm membrane filters (Jinteng, Tianjin, China). The supernatant was analyzed by high performance liquid chromatography (HPLC) analysis which were carried out on a Waters Alliance 2695 separation module with a 2998 photodiode array detector (Waters, MA, USA) and a Reversed-Phase 18 column (4.6mm × 250mm, 5 μm) (Agilent, CA, USA). The mobile phases were water with 0.1% phosphoric acid (A) and acetonitrile (B) with the flow rate of 1 mL/min. The injection volume is 10 μl, the column temperature was 30°C, and the detection wavelengths for CGA and luteoloside were 327 nm and 350 nm respectively. The gradient elution method was as follows: 0–2 min (12% B); 2–12 min (12%–20% B); 12–22 min (20% B); 22–47 min (20%–30% B).

### 2.9 Quantitative real-time PCR analysis

To validate the accuracy of the gene expression obtained from the RNA-Seq analysis, 22 genes associated with metal ion mediated signal transduction were selected for qRT-PCR. Primer 5.0 software was used to design primers (**Supplemental Table 1**) and qRT-PCR were conducted using ABI7500 fluorescence quantitative PCR instrument (Applied Biosystems, CA, USA). The SYBR^®^ Green Pro Taq HS qPCR Kit (Accurate Biotechnology Co., Ltd, Hunan, China) was used and three biological replicates were performed of each group sample. Relative expression levels were calculated based on the 2^− ΔΔCt^ method (Livak and Schmittgen, 2001) using actin as the house-keeping gene (Cai et al., 2022).

### 2.10 Statistical analysis

The SPSS statistical software (version 22.0; IBM, Armonk, NY, USA) was used for statistical evaluation. Statistical significance was evaluated by the Student’s *t*-test when only two groups were compared or one-way ANOVA followed by Tukey’s test when multiple groups were compared. A *p*-value< 0.05 was considered as the statistical significance. Three independent biological replicates per sample were tested in this study.

## 3 Results

### 3.1 Leaves morphology and physiology analyses of *L. japonica* under chilling treatment

In this study, the *L. japonica* plants were grown in a 10°C environment for 30 days. The leaves morphology had been monitored during the chilling phase. As it was identified that the color of leaves was changed into purple from green when the plants were treated with chilling for 15 days, and the purple color spread to the whole leaves when it reached 30 days (**Figure 1A**). The total chlorophylls, carotenoids, and anthocyanins were measured by using a spectrophotometric method (**Figure 1B**). The content of total chlorophylls was not significantly changed when the seedlings grown in both control and chilling treatment conditions. However, the content of carotenoids was dramatically increased in response to the chilling treatment that it was 84%-increase in leaves treated for 30 days compared with SP. Interestingly, the anthocyanins oppositely changed in the control and treatment groups. During the growth process, the content of anthocyanins slightly decreased under the control condition, however, it gradually increased under the chilling condition. The ratio of total chloropylls, carotenoids, and anthocyanins were calculated and located in the 3-dimensional diagram. **Figure 1C** shows that the locations of ratios at treatment for 15 days and 30 days were significantly changed.

### 3.2 Transcriptomic analysis of leaves under chilling treatment

**Figure 2:**
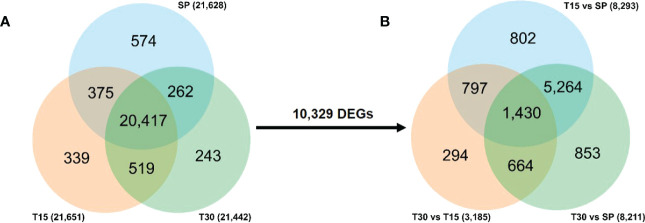
Venn diagram of transcriptomic data. **(A)** Venn diagram of totally identified genes; **(B)** Venn diagram of DEGs under chilling stress. The number on the arrow represents the DEGs among the co-identified genes in three samples.

To study the response mechanism of *L. japonica* to chilling treatment, leaves of *L. japonica* in the treatment group were collected for transcriptomic analysis. A total of 21,628, 21,651, and 21,442 genes were identified in leaves of *L. japonica* at SP, T15, and T30, respectively (**Figure 2**). During the identified genes, 20,417 genes were co-expressed among the three groups and 10,329 genes were differentially expressed under chilling stress. In **Figure 2**, it was shown that there were 8,293 and 8,211 genes that differentially expressed in response to T15 and T30. There were 3,185 genes differentially expressed when *L. japonica* were under chilling stress from 15 days to 30 days. Furthermore, the expression of 1,430 genes were significantly changed in response to both T15 and T30.

### 3.3 Function analysis of DEGs of *L. japonica* under chilling stress

**Figure 3:**
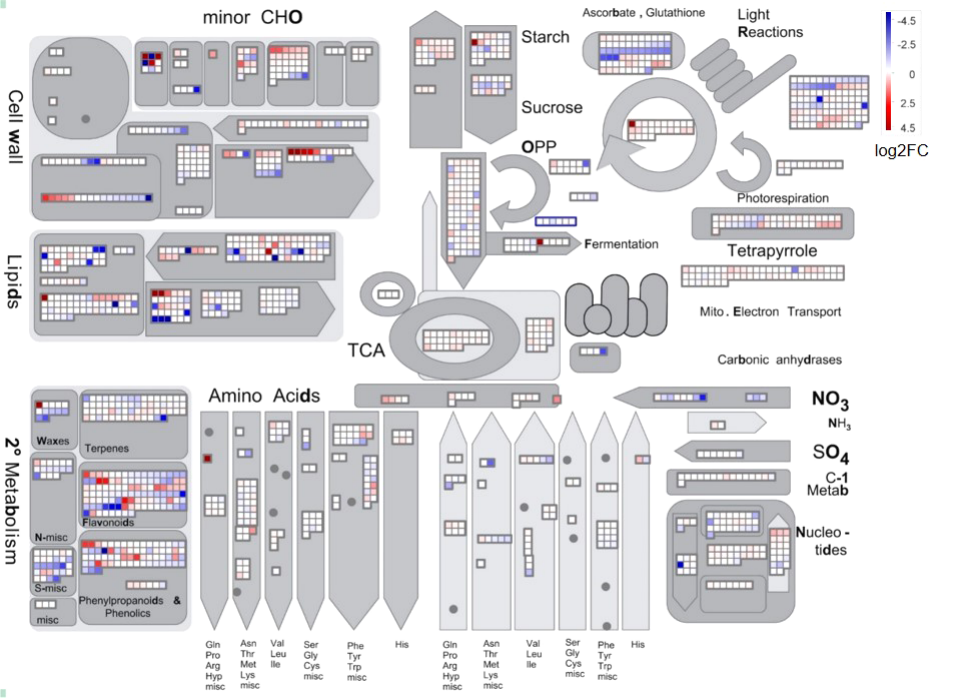
MapMan analysis of DEGs in *L. japonica* under chilling treatment for 30 days. The genes expression was compared between SP and T30. The Log2FC values of the gene expression were used for MapMan visualization using MapMan software to obtain an overview of the affected metabolic processes. Each colored square indicates the Log2FC value of a differentially changed gene. Red, blue, and white colors indicate an increase, decrease, and no change in the Log2FC value of gene expression.

To illustrate the effect of chilling stress on *L. japonica*, DEGs in *L. japonica* under chilling treatment for 30 days were functionally categorized using MapMan software (**Figure 3**). The mapped DEGs were mainly enriched in secondary metabolism, lipids, cell wall, and minor carbohydrate. The secondary metabolism was further dissected in **Supplemental Figures 1** and **2**. Most of the DEGs were involved in phenlypropanoids, flavonoids, and lignin/lignans. Very interestingly, genes related to non MVA pathway and sulfur containing metabolism were decreased in response to chilling stress, however, genes related to shikimate, chalcones, and isoflavonoids pathways were almost upregulated. It was further realized that genes in betains, simple phenols, and carotenoids were slightly upregulated in response to the stress. Anthocyanins, dihydroflavonols, flavonols related genes were differentially induced that they were upregulated and downregulated under the chilling stress. In the tetrapyrrole metabolism, although two genes involved in biosynthesis of chlorophyll a was upregulated, however, obviously, genes involved in biosynthesis of chlorophyll b and chlorphyllide a were downregulated in response to the chilling stress (**Supplemental Figure 3**).

### 3.4 Cluster analysis of expression patterns of DEGs

**Figure 4:**
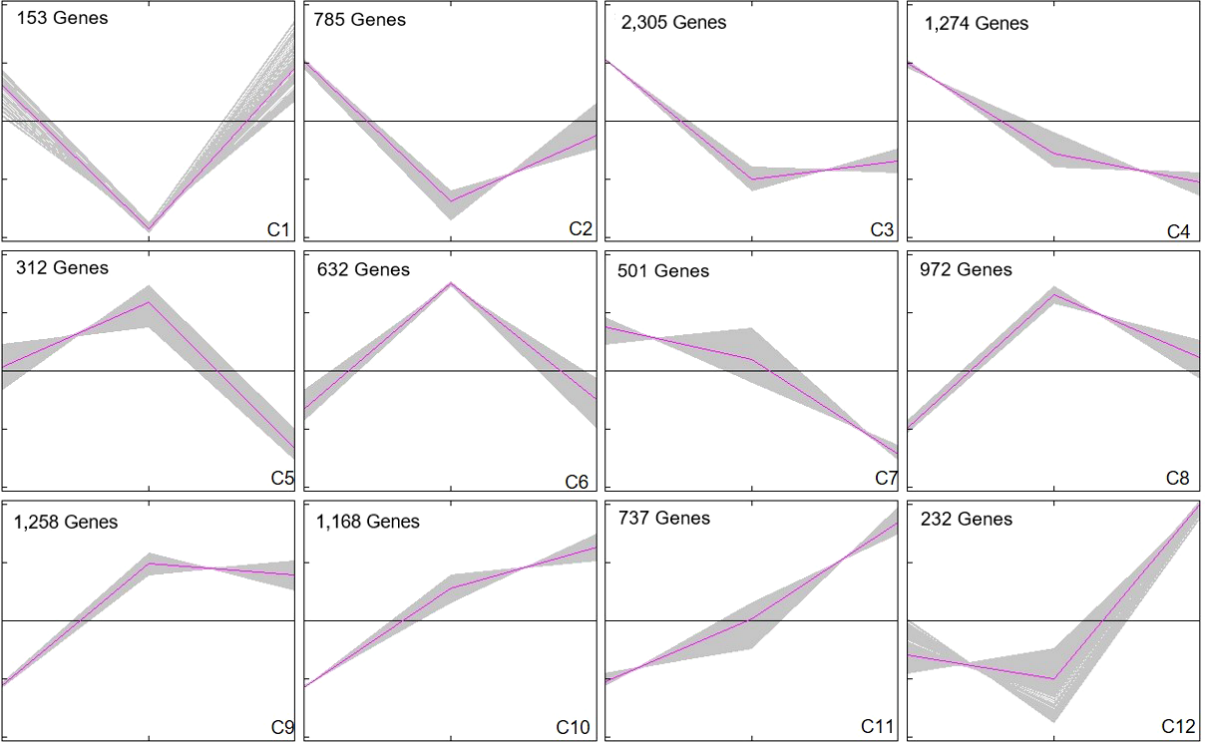
Cluster analysis of DEGs. DEGs were clustered and displayed in line chart using Mev. Twelve clusters were grouped. X-axis represents the time points during chilling stress including SP, T15, and T30. Y-axis represents normalized value of the DEGs expression level. The middle black line in each cluster is the zero line and the red line indicates the average expression level. The number in the upper left corner represents the number of DEGs in each cluster.

**Figure 5:**
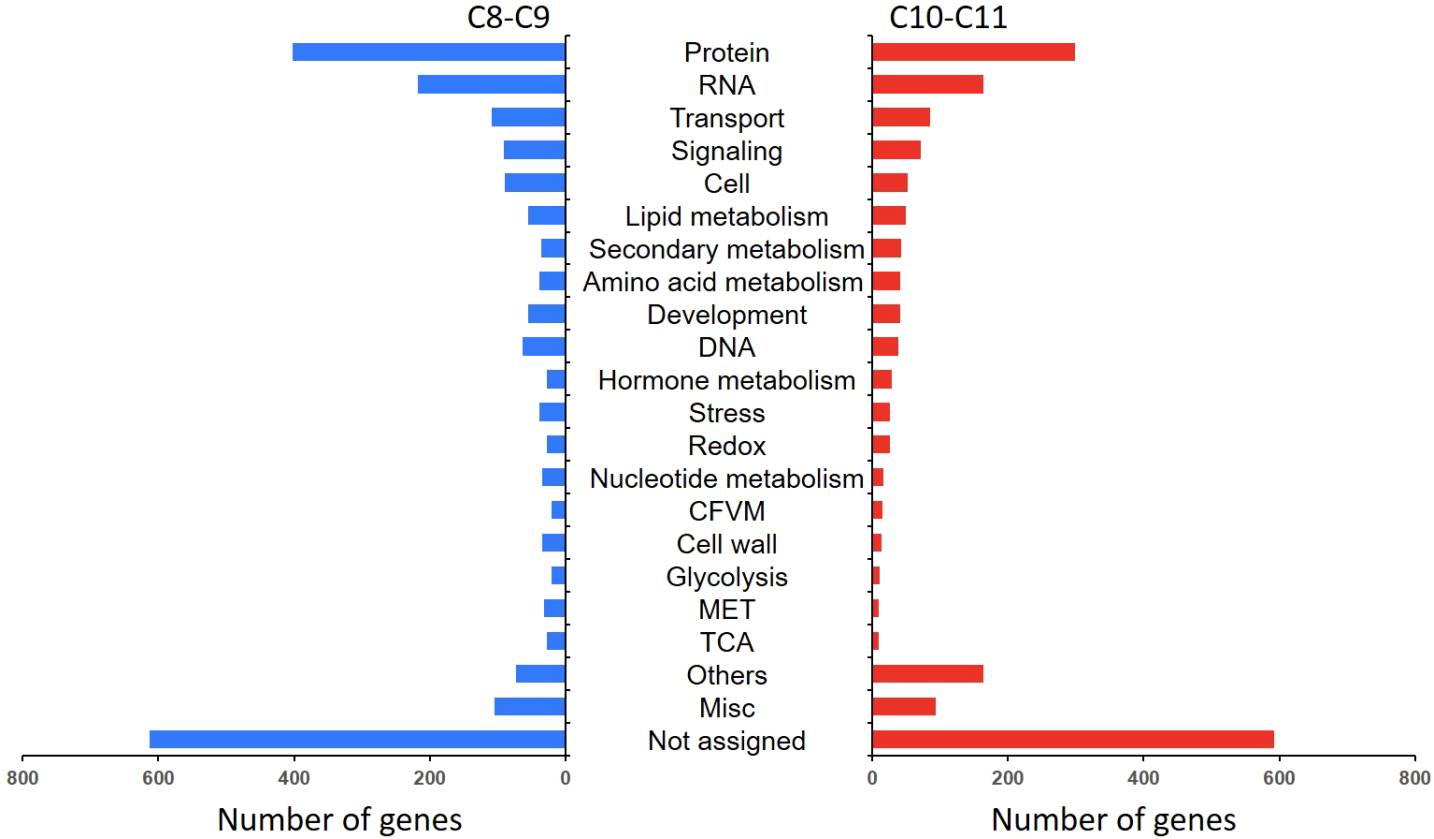
Functional category of DEGs in cluster 8 (C8), C9, C10, and C11. Gene function was predicted and categorized using MapMan bin codes. CFVM, Co-factor and vitamine metabolism; MET, mitochondrial electron transport; Others: Biodegradation of Xenobiotics, C1-metabolism, fermentation, major carbohydrate metabolism, metal handling, minor carbohydrate metabolism, oxidative pentose phosphate, polyamine metabolism, photosynthesis, S-assimilation, tetrapyrrole synthesis, and transporter.

In order to explore the response mechanism of *L. japonica* to chilling stress, DEGs were clustered based on their temporal expression profiles (**Figure 4**). Twelve clusters (C1-C12) which represented the different expression trends during the treatment process were generated by the K-means algorithm. Genes in clusters C1-C3 and C12 were downregulated at T15 while upregulated at T30. Conversely, genes in C5-C6 and C8-C9 were upregulated at T15 and downregulated at T30. Genes in C4 and C7 were gradually downregulated at T15 and T30. In C10-C11, genes were gradually upregulated at T15 and T30. Notablely, the expression of genes in C8-C11 were significantly higher at T15 and T30 than that at SP. Genes in clusters C8-C11 were further functionally analyzed. A total of 2,230 and 1,905 genes in C8-C9 and C10-C11, respectively, were homologously annotated by Arabidopsis genome and the function was categorized using MapMan bin codes (**Figure 5**). Both groups of genes were mainly enriched in protein metabolism, RNA, transport, signaling, cell metabolism, lipid metabolism, secondary metabolism, and amino acid metabolism. The number of genes enriched in nucleotide metabolism, cell wall, glycolysis, mitochondrial electron transport and TCA pathways was bigger in C8-C9 than in C10-C11.

### 3.5 PPI and functional category of transport and signaling related DEGs

**Figure 6:**
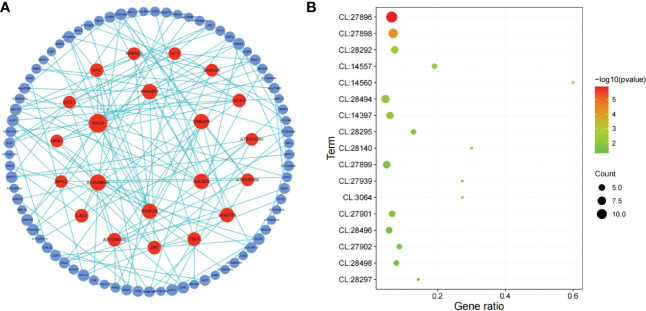
PPI analysis of DEGs related to signaling and transport in C10-C11. **(A)** Network of protein-protein interaction (PPI). The circles represent the proteins and the lines represent the interactions of two proteins. The size and color of circles indicate the degree value represented by the number of the interacting proteins. The circles with a high degree value (above 4) which was represented by the number of interacting proteins were pained by red color. **(B)** Bubble map of functional enrichment of genes. CL:27896, Mixed, incl. transition metal ion transport, and heavy metal-associated domain superfamily; CL:27898, Mixed, incl. transition metal ion transport, and heavy metal-associated domain superfamily; CL:28292, Calcium transport, and potassium ion transport; CL:14557, Mixed, incl. citrate transporter, and tlc atp/adp transporter; CL:14560, Citrate transporter, and plasma membrane pyruvate transport; CL:28494, Mixed, incl. anion transmembrane transporter activity, and lateral organ boundaries, lob; CL:14397, Mixed, incl. chloroplast membrane, and mitochondrial carrier protein; CL:28295, Sodium/calcium exchanger protein, and Cation transporting ATPase, C-terminus; CL:28140, Mixed, incl. abc transporter, cbio/ecfa subunit, and ferric-chelate reductase activity; CL:27899, Mixed, incl. transition metal ion transmembrane transporter activity, and heavy metal-associated domain superfamily; CL:27939, Mixed, incl. iron transport, and nickel transport; CL:3064, Mixed, incl. gtp-binding protein enga, and endoplasmic reticulum vesicle transporter; CL:27901, Mixed, incl. zinc ion transmembrane transport, and iron ion homeostasis; CL:28496, Mixed, incl. nitrate assimilation, and malate transport; CL:27902, Mixed, incl. iron ion homeostasis, and nicotianamine synthase; CL:28498, Mixed, incl. response to nitrate, and molybdenum cofactor biosynthesis; CL:28297, Sodium/calcium exchanger protein, and Cation transporting ATPase, C-terminus.

**Figure 5** shows that most DEGs in *L. japonica* under chilling stress were involved in transport and signaling processes. In order to further reveal the response mechanism of *L. japonica* to chilling treatment, DEGs related to transport and signaling processes in group C10-C11 were selected for protein-protein interaction analysis. In **Table 1**, 15 and 13 DEGs function as the metabolite transporters at the mitochondrial membrance and ABC transporters/multidrug resistance system, respectively, belonging to transport process, while there are 22 and 20 DEGs function as receptor kinases and G-proteins, respectively, belonging to signaling process. PPI analysis told that 21 genes were more connected with other genes based on their interaction in the database (**Figure 6A**). By using the local network cluster method, 17 clusters were produced with a false discovery rate below 0.05. The top three enrichment functions are transition metal ion transport (CL:27896 and CL:27898) and calcium transport/potassium ion transport (CL:28292) (**Figure 6B**). There are 6 genes including *NCL*, tonoplast Na+/H+ antiporter 2 (*NHX2*), NCRK, ACA9, CAX3, autoinhibited Ca (2+) ATPases 8 (*ACA8*) gathered in cluster of CL:28292. Interestingly, four of the six genes (*NCL*, *ACA9*, *CAX3*, and *ACA8*) concurrently work as sodium or calcium exchanger protein/cation transporting ATPase/C-terminus.

**Table 1:** Genes involved in processes of transport and signaling.

no	Gene ID^a^	Description	Primary_function^b^	Secondary_function^c^
1	Lj9C504G12	pleiotropic drug resistance 12	transport	aBC T/M resistance systems
2	Lj4C799T6	p-glycoprotein 13	transport	aBC T/M resistance systems
3	Lj8A190G37	aBC transporter family protein	transport	aBC T/M resistance systems
4	Lj3A1058G56	aTTAP1	transport	aBC T/M resistance systems
5	Lj4A0G28	aBC1 family protein	transport	aBC T/M resistance systems
6	Lj2A115G52	aTNAP12	transport	aBC T/M resistance systems
7	Lj2C115T13	aTNAP12	transport	aBC T/M resistance systems
8	Lj1P929T42	aBC transporter family protein	transport	aBC T/M resistance systems
9	Lju99C7G4	pleiotropic drug resistance 9	transport	aBC T/M resistance systems
10	Lj6A677T75	aTMRP10	transport	aBC T/M resistance systems
11	Lj7A748T61	aBC1 family protein	transport	aBC T/M resistance systems
12	Lj2C606G6	aTNAP13	transport	aBC T/M resistance systems
13	Lj5A173G70	antiporter	transport	aBC T/M resistance systems
14	Lj2A1105G55	STARIK 1	transport	aBC T/M resistance systems
15	Lj1A219T39	amino acid transporter family protein	transport	amino acids
16	Lj4A229T23	amino acid transporter family protein	transport	amino acids
17	Lj2P342T86	Cationic amino acid transporter 5	transport	amino acids
18	Lj2A72T46	CAX-interacting protein 2	transport	Calcium
19	Lj9C159G2	autoinhibited Ca2+-ATPase,isoform 8	transport	Calcium
20	Lju50A42T21	autoinhibited Ca2+-ATPase,isoform 8	transport	Calcium
21	Lju50A4G44	autoinhibited Ca2+-ATPase,isoform 8	transport	Calcium
22	Lj2A356T43	Ca2+-ATPase, isoform 8	transport	major Intrinsic Proteins
23	Lj8A252G39	ozone-sensitive 1	transport	metabolite transporters at TMM
24	Lj5A59G64	bAC2	transport	metabolite transporters at TMM
25	Lj5C171G24	mitochondrial substrate carrier family protein	transport	metabolite transporters at TMM
26	Lj1A922T71	mitochondrial substrate carrier family protein	transport	metabolite transporters at TMM
27	Lj2A76T76	binding/transporter	transport	metabolite transporters at TMM
28	Lj3A954G61	binding/transporter	transport	metabolite transporters at TMM
29	Lj3A911T23	mTM1	transport	metabolite transporters at TMM
30	Lj4C863G12	s-adenosylmethionine carrier 1	transport	metabolite transporters at TMM
31	Lj1A331T23	dicarboxylate transporter 1	transport	metabolite transporters at TMM
32	Lj4C121T24	mitochondrial substrate carrier family protein	transport	metabolite transporters at TMM
33	Lju2039C0T1	mitochondrial substrate carrier family protein	transport	metabolite transporters at TMM
34	Lj3A838T62	mitochondrial substrate carrier family protein	transport	metabolite transporters at TMM
35	Lj7P367T38	mitochondrial substrate carrier family protein	transport	metabolite transporters at TMM
36	Lj8A300T30	a bout de souffle	transport	metabolite transporters at TMM
37	Lj9C349T1	dicarboxylate transport 2.1	transport	metabolite transporters at TMM
38	Lj7A85T50	natural resistance-associated macrophage protein 3	transport	metal
39	Lj9P608T13	Zinc transporter of arabidopsis thaliana	transport	metal
40	Lj5A213T83	aTNHD1	transport	metal
41	Lj3A803G20	Cobalt ion transmembrane transporter	transport	metal
42	Lj8A59T32	Cation/H+ exchanger 4	transport	metal
43	Lj2A75T36	Cation exchanger 3	transport	metal
44	Lju857A2T20	metal tolerance protein	transport	metal
45	Lj4A156G48	Calcium-transporting ATPase	transport	metal
46	Lj5C328G3	iron-regulated protein 3	transport	metal
47	Lj1A1025T76	yellow stripe like 3	transport	metal
48	Lj3C901T9	yellow stripe like 3	transport	metal
49	Lj8A201T87	yellow stripe like 3	transport	metal
50	Lj3A1057T78	mATE efflux family protein	transport	misc
51	Lj1C899G5	SEC14 cytosolic factor family protein	transport	misc
52	Lj4A812T70	auxin efflux carrier family protein	transport	misc
53	Lj6C606T6	integral membrane transporter family protein	transport	misc
54	Lj9A197T42	xanthine/uracil permease family protein	transport	misc
55	Lj1A1229T46	mATE efflux family protein	transport	misc
56	Lj9A725G33	aTG18B	transport	misc
57	Lj5A126G85	transporter	transport	misc
58	Lj6A794T102	arabidopsis thaliana nitrate transporter 1:2	transport	nitrate
59	Lj7A596T39	arabidopsis thaliana high affinity nitrate transporter 2.7	transport	nitrate
60	Lj9A484G61	de-etiolated 3	transport	p- and v-ATPases
61	Lj2C511T3	ala-interacting subunit 1	transport	p- and v-ATPases
62	Lj5C57T5	proton-dependent oligopeptide transport family protein	transport	peptides and oligopeptides
63	Lj7C704G4	proton-dependent oligopeptide transport family protein	transport	peptides and oligopeptides
64	Lj6A760G42	proton-dependent oligopeptide transport family protein	transport	peptides and oligopeptides
65	Lj1C1084T2	proton-dependent oligopeptide transport family protein	transport	peptides and oligopeptides
66	Lj3C1042T8	proton-dependent oligopeptide transport family protein	transport	peptides and oligopeptides
67	Lj2A80T61	peptide transporter 1	transport	peptides and oligopeptides
68	Lj7A581T56	peroxisomal membrane protein 36	transport	peroxisomes
69	Lj6C784G16	EXS family protein	transport	phosphate
70	Lj1A141T51	pHT4	transport	phosphate
71	Lj6A74T31	voltage dependent anion channel 1	transport	porins
72	Lj6A8G17	voltage dependent anion channel 1	transport	porins
73	Lj7C544G3	KUP6	transport	potassium
74	Lj5C239G18	KUP7	transport	potassium
75	Lj9A353G67	KUP7	transport	potassium
76	Lj1C1187T1	KUP7	transport	potassium
77	Lj8A178T71	potassium channel in Arabidopsis thaliana 1	transport	potassium
78	Lj9C319T7	SULTR1	transport	Sulphate
79	Lj7C436G16	Chloride channel-like (CLC) protein	transport	unspecified anions
80	Lj2A17G38	anion-transporting ATPase family protein	transport	unspecified anions
81	Lj3A357T51	Sodium symporter-related	transport	unspecified cations
82	Lj3A376T54	Sodium symporter-related	transport	unspecified cations
83	Lj3C357T11	Sodium symporter-related	transport	unspecified cations
84	Lj3C376T8	Sodium symporter-related	transport	unspecified cations
85	Lj6C668T12	Sodium hydrogen exchanger 2	transport	unspecified cations
86	Lj1A101G44	bile acid:sodium symporter family protein	transport	unspecified cations
87	Lj4C8G7	Calcium-binding EF hand family protein	signaling	Calcium
88	Lj8A152T84	Calcium exchanger family protein	signaling	Calcium
89	Lj2C387T4	Calcium-binding EF hand family protein	signaling	Calcium
90	Lj9A541T96	autoinhibited Ca(2+)-ATPase 9	signaling	Calcium
91	Lj3C929T10	Calmodulin-binding family protein	signaling	Calcium
92	Lj3P939T25	Calcium-binding protein	signaling	Calcium
93	Lj2A113T37	Synaptotagmin-3-like isoform X1	signaling	Calcium
94	Lj3C920T4	Calcium dependent protein kinase 1	signaling	Calcium
95	Lj1A329T70	Calmodulin-domain protein kinase 7	signaling	Calcium
96	Lj1C329T2	Calcium-dependent protein kinase 19	signaling	Calcium
97	Lj5A158T70	zinc finger (Ran-binding) family protein	signaling	G-proteins
98	Lj1C905T5	GTP-binding protein-related	signaling	G-proteins
99	Lju124C44G2	GTP-binding family protein	signaling	G-proteins
100	Lj1C332G7	Embryo defective 2738	signaling	G-proteins
101	Lj2A373G38	rab GTPase homolog a4d	signaling	G-proteins
102	Lj2A1172G33	GTP-binding family protein	signaling	G-proteins
103	Lj1A40T79	rac GTPase activating protein	signaling	G-proteins
104	Lj4A705T44	Ras-related GTP-binding protein	signaling	G-proteins
105	Lj7C386T22	Ras-related GTP-binding protein	signaling	G-proteins
106	Lj2A1147T78	RabGAP/TBC domain-containing protein	signaling	G-proteins
107	Lj4A222T52	arabidopsis rac-like 6	signaling	G-proteins
108	Lj4A817G44	GTP-binding protein LepA	signaling	G-proteins
109	Lj4C817T5	GTP-binding protein LepA	signaling	G-proteins
110	Lj4A817T44	GTP-binding protein LepA	signaling	G-proteins
111	Lj5A87T64	Scarface	signaling	G-proteins
112	Lj2C416G9	variegated 3	signaling	G-proteins
113	Lj3C814G4	GTP1/OBG family protein	signaling	G-proteins
114	Lj7C649T3	rab homolog 1	signaling	G-proteins
115	Lj8C620T2	ran GTPase 3	signaling	G-proteins
116	Lj4A33G81	GTP-binding family protein	signaling	G-proteins
117	Lj2C43G5	Chloroplastic NIFS-like cysteine desulfurase	signaling	in sugar and nutrient physiology
118	Lj2A615G13	Glucose-inhibited division family A protein	signaling	in sugar and nutrient physiology
119	Lj2A615T20	Glucose-inhibited division family A protein	signaling	in sugar and nutrient physiology
120	Lj5A721G18	intracellular ligand-gated ion channel	signaling	in sugar and nutrient physiology
121	Lj5A725G36	intracellular ligand-gated ion channel	signaling	in sugar and nutrient physiology
122	Lj5C720T4	intracellular ligand-gated ion channel	signaling	in sugar and nutrient physiology
123	Lj5C727G2	intracellular ligand-gated ion channel	signaling	in sugar and nutrient physiology
124	Lj5C720G5	intracellular ligand-gated ion channel	signaling	in sugar and nutrient physiology
125	Lj5A66T60	phototropic-responsive NPH3 family protein	signaling	light
126	Lj3A964T54	interPro: IPR018618	signaling	light
127	Lj1A24T62	photolyase/blue-light receptor 2	signaling	light
128	Lj6P646T44	Early light-inducable protein	signaling	light
129	Lj1A1175T55	binding/catalytic/transcription repressor	signaling	light
130	Lj4A34T50	arabidopsis thaliana mitogen-activated protein kinase homolog 2	signaling	mAP kinases
131	Lju124C9T0	arabidopsis thaliana nudix hydrolase homolog 26	signaling	phosphinositides
132	Lj5C221T8	inositol 1,3,4-trisphosphate 5/6-kinase family protein	signaling	phosphinositides
133	Lj9A306T61	phosphatidylinositol-4-phosphate 5-kinase family protein	signaling	phosphinositides
134	Lj2A1082T56	phosphoinositide-specific phospholipase C family protein	signaling	phosphinositides
135	Lj1C1161T14	protein kinase family protein	signaling	receptor kinases
136	Lj8C92T9	protein kinase	signaling	receptor kinases
137	Lj2C1140G2	abnormal Leaf Shape 2	signaling	receptor kinases
138	Lj9C344T8	lectin protein kinase	signaling	receptor kinases
139	Lj9C516T6	light repressible receptor protein kinase	signaling	receptor kinases
140	Lj6A740G47	aTP binding/kinase/protein serine/threonine kinase	signaling	receptor kinases
141	Lj6C733T14	transmembrane kinase 1	signaling	receptor kinases
142	Lj1C1210T6	Strubbelig-receptor family 3	signaling	receptor kinases
143	Lj1C734G1	Strubbelig-receptor family 3	signaling	receptor kinases
144	Lj2C371T5	Strubbelig-receptor family 2	signaling	receptor kinases
145	Lj1C112T13	leucine-rich repeat transmembrane protein kinase	signaling	receptor kinases
146	Lj4A746T58	LRR XI-23	signaling	receptor kinases
147	Lj4P546T31	leucine-rich repeat transmembrane protein kinase	signaling	receptor kinases
148	Lj4C170T6	HAESA-Like 2	signaling	receptor kinases
149	Lj3P711T17	leucine-rich repeat transmembrane protein kinase	signaling	receptor kinases
150	Lj9A444G106	protein kinase family protein	signaling	receptor kinases
151	Lj3C1059T10	Chloroplast sensor kinase	signaling	receptor kinases
152	Lj1A202G34	nCRK	signaling	receptor kinases
153	Lj3C851G6	protein kinase	signaling	receptor kinases
154	Lj1A858G108	protein kinase	signaling	receptor kinases
155	Lj8A162T65	protein kinase	signaling	receptor kinases
156	Lj2C125T0	protein kinase-related	signaling	receptor kinases
157	Lj7A225T45	mechanosensitive channel of small conductance-like 10	signaling	unspecified

a Gene ID, gene location in the genome of L. japonica, ^b, c^Function, annotation by Mapman bin codes.

### 3.6 Pathway mapping of secondary metabolism related DEGs in *L. japonica* under chilling stress

**Figure 7:**
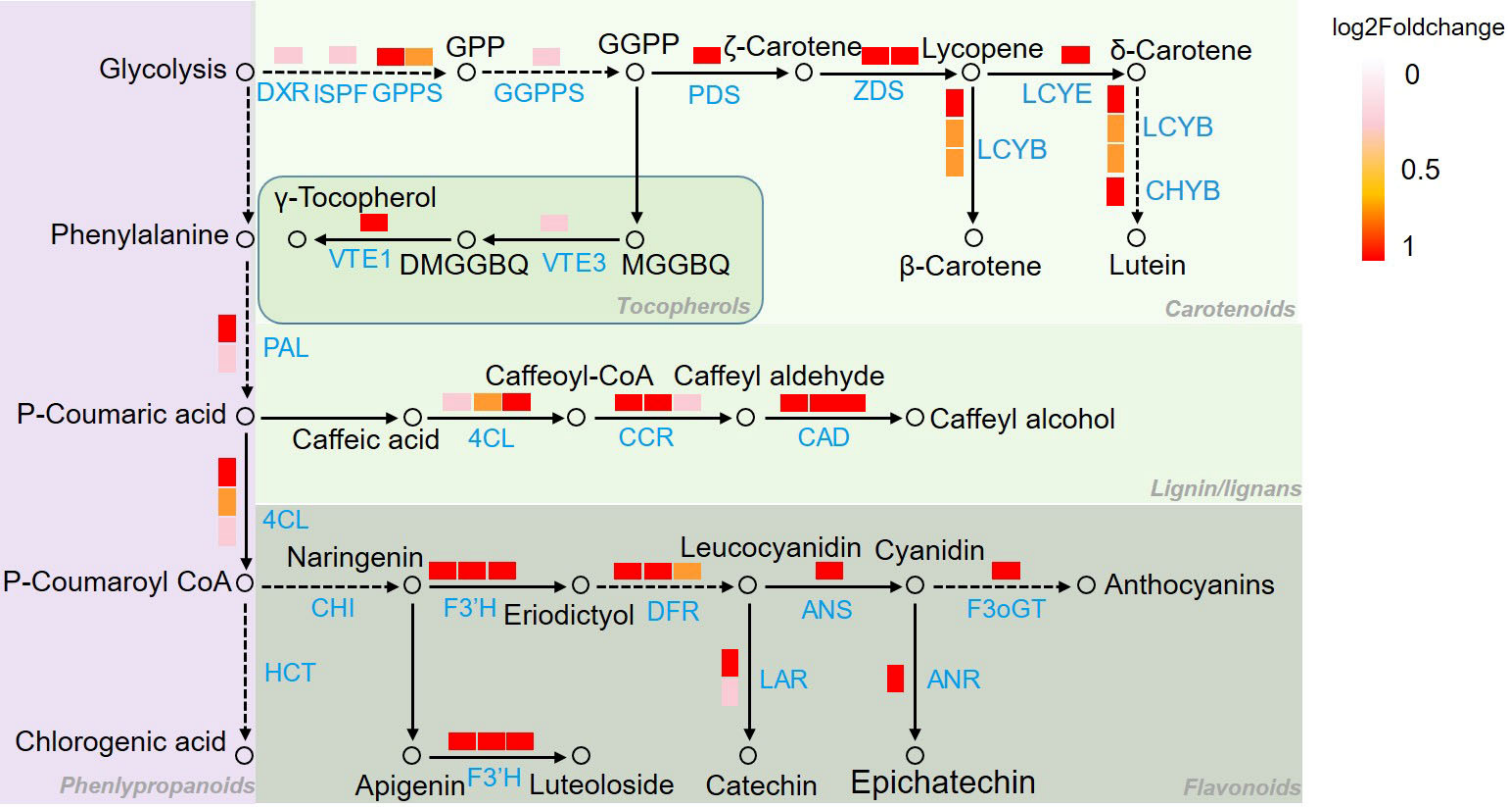
Pathway mapping of secondary metabolism related DEGs in **Figure 5**. Genes identification and expression were represented by boxes with different color. Metabolites were represented by hollow circles. 4CL, 4-coumarate: CoA ligase; ANR, anthocyanidin reductase; ANS, anthocyanidin synthase; CAD, cinnamol dehydrogenase; CCR, cinnamoyl CoA reductase; CHS, chalcone synthase; LCYB, lycopene beta cyclase; LCYE, lycopene epsilon cyclase; CHYB, β-carotene hydroxylase, F3’H, flavonoid 3’-hydroxylase; DFR, dihydroflavonol 4-reductase, DXR, 1-deoxy-D-xylulose 5-phosphate reductase; F3oGT, flavonol-3-O-glucosyl transferase; GGPPS, geranylgeranyl pyrophosphate synthase; GPPS, geranyl pyrophosphate synthase; ISPF, 2C-methyl-d-erythritol 2,4-cyclodiphosphate synthase; LAR, leucoanthocyantin reducase, PAL, phenylalanine ammonia lyase; PDS, phytoene desaturase; VTE1, tocopherol cyclase; VTE3, 2-methyl-6-phytyl-1,4-benzoquinol methyltransferase; ZDS, ζ-carotene dehydrogenase; DXP, 1-deoxy-D-xylulose 5-phosphate; MEcPP, 2-C-methyl-d-erythritol-2,4-cyclopyrophosphate; MEP, 2-C-methyl-D-erythrifol 4-phosphat; DMGGBQ, 6-geranylgeranyl-2,3-dimethylbenzene-1,4-diol, MGGBQ, 6-geranylgeranyl-2-methylbenzene-1,4-diol.

To illustrate the response mechanism of secondary metabolism to chilling stress, secondary metabolism related genes in C8-C9 and C10-C11 were mapped to KEGG database. As it was shown in **Figure 7**, genes of PAL and 4CL which are related to CGA biosynthesis involved in phenylalanine metabolism were upregulated under chilling stress. In the α-tocopherol biosynthetic pathway, VTE1 and VTE3 were together upregulated in reponse to the stress. Furthermore, DXR, ISPF, GGPS, and GGPPS in the geranylgeranyl-PP biosynthetic pathway, with PDS, ZDS, LCYE, LCYB in the β-carotene biosynthetic pathway were significantly activated by the chilling stress. Another obvious cue is the activation of luteolin biosynthetic pathway based on the upregulation of CHS and CYP75B1. Moreover, genes of F3’H, DFR, LAR, ANS, ANR, and F3oGT which function in the synthetic process of anthocyanins were also remarkably induced by the chilling stress. Additionally, CCoAR and CAD involved in lignins biosynthetic pathway were found to be upregulated in response to chilling stress.

### 3.7 Analysis of chlorogenic acid and luteoloside content

**Figure 8:**
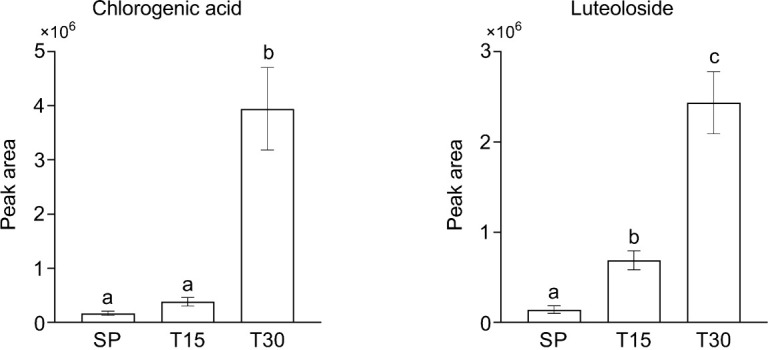
The content of chlorogenic acid and luteoloside in samples. Leaves of *L. japonica* were collected at SP, T15, and T30. CGA and luteoloside were extracted by methanol and analyzed by HPLC. Data are shown as mean ± S.D. from three independent biological replicates. Different lowercase letters indicate statistically significant differences as measured by Tukey’s test (p< 0.05).

PPI analysis proved that DEGs with more connections in both categories of transport and signaling participate in calcium transport process. Pathway mapping of secondary metabolism related DEGs indicated the activation of biosynthetic pathways of CGA and luteoloside. CGA and luteoloside content were measured by HPLC to reveal the effect of chilling stress on secondary metabolism of *L. japonica* leaves. **Figure 8** shows that CGA and luteoloside were both gradually accumulated from SP to T30. At T30, the content of two metabolites had a dramatic increase that they were 23 and 17 folds compared with those at SP.

### 3.8 Phylogenetic analysis

**Figure 9:**
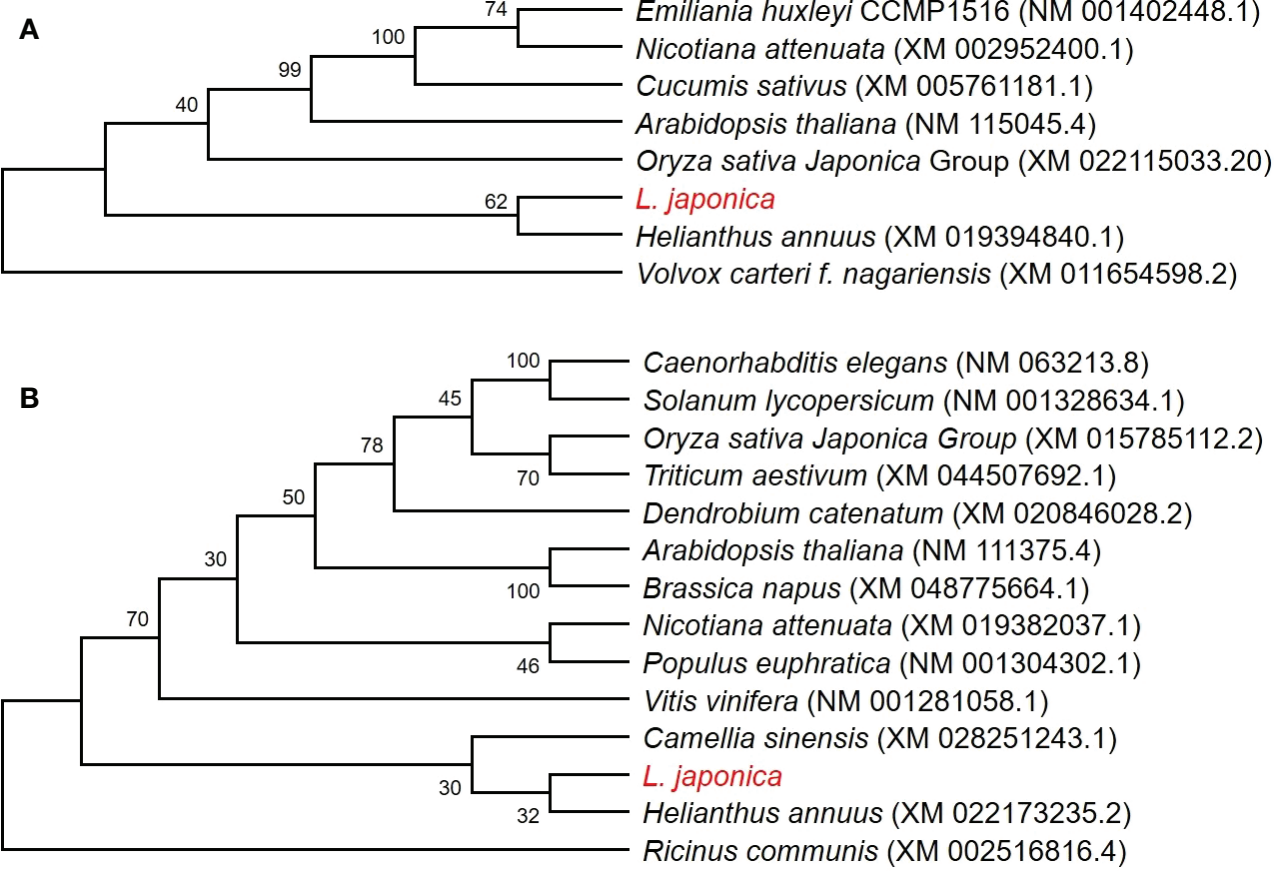
Phylogenetic analyses of *CAX3* and *NHX2* in *L. japonica* and other species. Phylogenetic trees were generated using *CAX3* of *L. japonica* with other 7 species **(A)**, and *NHX2* of *L. japonica* with other 13 species **(B)**, respectively. The numbers at nodes in the phylogenetic tree indicate bootstrap values per 1000 replicates. The coding sequences were obtained from NCBI and the source of data is noted after species names.

Two Neighbor-Joining phylogenetic trees were constructed using 8 *CAX3* genes and 14 *NHX2* genes, respectively, to explore the evolutionary relationship between *L. japonica* and other selected species. Based on the nucleotide sequences of *CAX3* in the species, it is learned that *L. japonica* is relatively closed to *Helianthus annuus*, while they are evolutionarily distant to *Emiliania huxleyi CAX3*, *Nicotiana attenuate CAX3*, *Cucumis sativus CAX3*, and *Arabidopsis thaliana CAX3*, which are highly homologous (**Figure 9A**). However, a lower evolutionary relationship is found between *L. japonica NHX2* and *Helianthus annuus NHX2* than *CAX3* (**Figure 9B**). Interestingly, it seems that the evolution of *NHX2* genes is not conservative among these species.

### 3.9 Gene expression analysis

**Figure 10:**
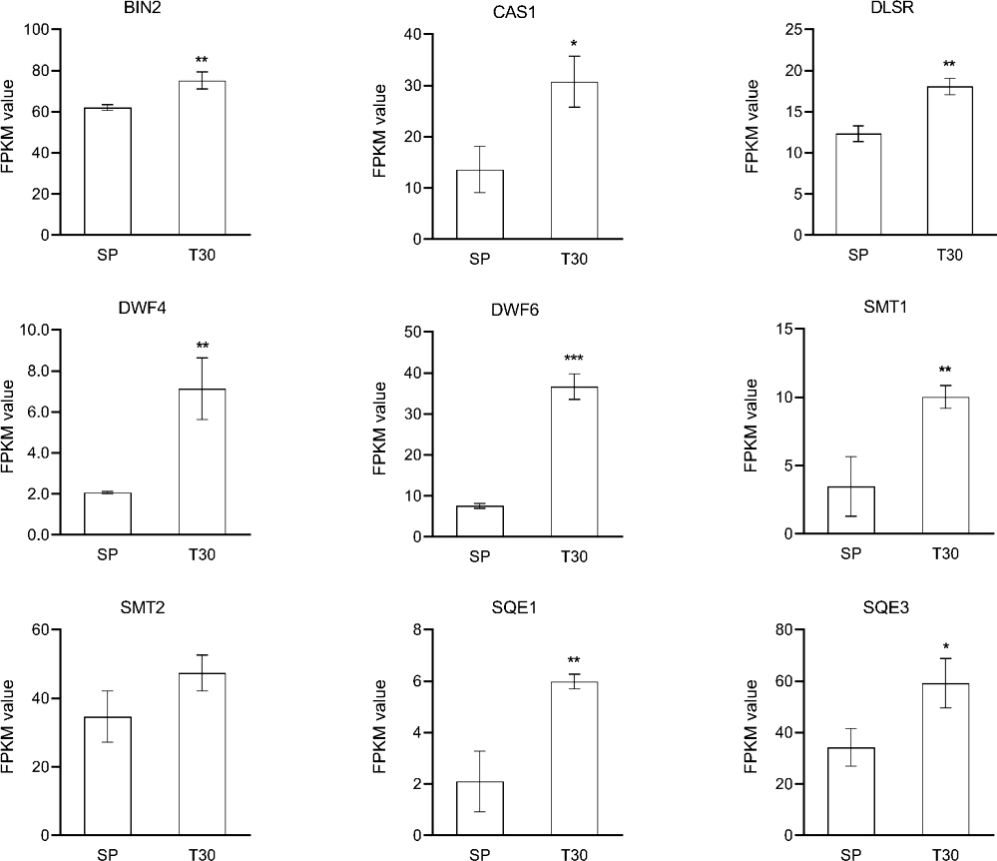
Expression analysis of BR related genes. The expression level of genes was represented by FPKM value. Data are represented as the mean ± S.D. from 3 independent biological replicates. Means with the " * " were significantly different among samples according to one-way ANOVA test ("*", p< 0.05; "**", p< 0.01; "***", p< 0.001). BIN, brassinosteroid-insensitive; CAS1, cycloartenol synthase 1; DLSR, delta14-sterol reductase; SMT1, sterol methyltransferase 1; SMT2, sterol methyltransferase 2; SQE1, squalene epoxidase1; SQE3, squalene epoxidase 3.

Based on the protein interaction results in **Figure 6**, 22 genes potentially associated with metal ion-mediated signaling were screened for qRT-PCR (**Supplemental Figure 4**). The results showed that compared with SP, the expression of the 22 genes under chilling stress was significantly upregulated, which is consistent with the results of transcriptome analysis. In the brassinosteroids (BR) pathway, *DWF4*, *FK*, *SQE1*, *SQE3*, *SMT1*, *DETS*, and *CAS1* were collectively induced to a higher transcription level by chilling stress (**Figure 10**). Particularly, the dramatic regulation of chilling stress was identified on the expression of *DWF4* and *DET2*. Furthermore, as the receptor protein of BR, the transcription expression of BRI was also elevated under the chilling stress.

## 4 Discussion

### 4.1 Chilling stress activated the biosynthesis of flavonoids and carotenoids

Low temperature is one of the common abiotic stresses affecting plant growth. Plants adapt to low temperatures by changing their morphology and adjusting the expression of a series of genes involved in complex networks (Lantzouni et al., 2020; Li et al., 2021a). As an interesting phenotype response to chilling stress, leaves color changes have been concerned in many previous studies (Ahmed et al., 2015). It is suggested that, in most plants, the accumulation and composition of three pigments including chlorophylls, carotenoids, and anthocyanins determine the external expression of leaves color (Markwell and Namuth, 2003). When *Brassica campestris* L. was under low temperature, the leaves color was changed from green to yellow might contribute to the regulation of the pigments by *HY5* and its downstream genes (Yuan et al., 2021). It was proved that the leaves of Chinese cabbage turned purple under the low temperature stress with the increased accumulation of anthocyanin (Dai et al., 2022). In this study, the leaves of *L. japonica* were predictably changed into purple when under chilling condition. Here, our experiments not only suggested the acute accumulation of anthocyanins and carotenoids, but also proved that the ratio of total chloropylls, carotenoids, and anthocyanins was significantly changed in response to chilling stress. The upregulated genes (UGs) in response to chilling stress were functionally enriched and secondary metabolism related genes were well mapped to anthocyanins and carotenoids biosynthetic processes (**Figure 7**). Additionally, the pathway mapping of UGs and HPLC analysis proved that the chilling stress also increased the content of CGA and luteoloside (**Figure 8**). As the two compounds are suggested to be the index components for evaluating the quality of *L. japonica*, our study indicates that chilling stress has potential application in improving the medicinal quality and enhancing the ornamental value of *L. japonica*.

### 4.2 The homoeostasis of Ca^2+^ was reached a high level in leaves under chilling stress

The comparative transcriptomic strategy was conducted to demonstrate the molecular mechanism underlying the morphological response of *L. japonica* to chilling stress. The UGs were functionally categorized by MapMan bin codes and interacted by PPI software. Transport and signaling related genes were very positively induced to respond to chilling stress (**Figure 5**). Further analysis demonstrated that the interacted genes function in transition metal ion transport (**Figure 6**). It has been proved that Ca^2+^ level could be rapidly induced and increased in cytoplasm by cold stress (Liu et al., 2021). In melatonin treated Arabidopsis, Ca^2+^ efflux was induced accompanied by an increase of *CAX3*, and the *CAX3* deletion resulted in decreased Ca^2+^ efflux (Li et al., 2021b). In *L. japonica*, the transcript level of *CAX3* was detected to significantly increased under chilling stress, might indicating the rapid accumulation of calcium content. However, it is unknown whether calcium channels are involved in temperature sensing and how the Ca^2+^ signal is induced and decoded in response to chilling stress in *L. japonica*. Plants employee a combination of ion pumps, antiporters, and uniporters to control cytoplasmic calcium dynamics (Sanders et al., 2002) including a family of calmodulin-activated Ca^2+^-ATPase ion pumps like autoinhibited Ca^2+^ ATPases (ACAs) for Ca^2+^ transporter (Schiøtt et al., 2004). *ACA8* is suggested to be as a prominent regulator of Ca^2+^ dynamics (Bonza et al., 2000). *NCX* family members play important roles in mediating the Ca^2+^ homeostasis of plant under stress environment. In response to temperature decrease, *NCX* elevates intracellular Ca^2+^, which activates Ca^2+^/calmodulin-dependent protein kinase II and accelerates transcriptional oscillations of clock genes (Kon et al., 2021). Moreover, NHXs was reported to have a role in Na+ uptake mechanisms and transport pathway (Shavrukov, 2014), by which the plants could mediate Na+ uptake and compartmentation from/into the vacuole to mediate the homoeostasis under salt tolerance. It was documented that the stimulation of dynamic Ca^2+^ level on MHX for its proton signaling is a conserved regulation mechanism (Allman et al., 2013). In our study, whatever, the increased transcriptional level of identified *CAX3*, *NCX*, *NHX2*, and *ACA8* indicated chilling stress led to a high level homoeostasis of Ca^2+^ through improvement of ion transporting in leaves of *L. japonica*.

### 4.3 BR signaling pathway was induced by Ca^2+^ signaling and responsible for the accumulation of flavonoids and carotenoids

Although the complex regulation network of plants responding to chilling stress has not been fully elucidated (Ding et al., 2020), the accumulating evidences were located at the iron transporter as a cold sensor to initiate multiple responses (Zhang et al., 2019). The spraying of calcium induced the activation of flavonoid metabolism in grape (Zhang et al., 2021). The homeostasis of Ca^2+^ across the plasma membrane is critical for coordination of the downstream responses, suggesting a mechanistic link between the receptor complex and signaling kinases *via* Ca^2+^ as the secondary messenger. In Arabidopsis, *ACA8* interacts with brassinosteroid insensitive 1 (BRI1) to regulate plant physiology (Schwessinger et al., 2011). It is known that BRI1 is one of the key positive regulators of BR signaling (Clouse, 2011). Publications have shown that BR enhances stress tolerance and prevents cellular damage by abiotic environmental conditions (Bajguz and Hayat, 2009). Studies illustrated that BR signaling differentially affected plant flavonoid biosynthesis depending on the downstream regulators. BR accelerated the induced flavonoid accumulation by JA in Arabidopsis (Peng et al., 2011) and nitric oxide in *Camellia sinensis* L. (Li et al., 2017) through regulating the relative gene expression. Alternatively, BR signaling was found to inhibit the flavonoid biosynthesis through repressing the expression of MYB11, MYB12, and MYB111 by BR1-EMS-Suppressor 1 (Liang et al., 2020). In fact, it has been realized that studies are very limited on explore the complex and exact regulation mechanism of BR signaling on flavonoids biosynthesis, especially under an extreme temperature like chilling stress. As it was detected that one of the genes annotated as MYB111 was upregulated in leaves of L. japonica in response to chilling stress (**Supplemental Table 2**). Moreover, evidence supplied for an attached effect of BR signaling that the overexpression of BRI1 in tomato enhanced the endogenous BR signaling intensity and increased the carotenoids production (Nie et al., 2017). In this study, the KEGG enrichment of DEGs showed that the steroid hormone biosynthesis pathway exhibited very positive response to the cold stress. *BRI*, *DWF4*, *DLSR*, and *SMT2* were all upregulated, which were responsible for the biosynthesis of BR in leaves of *L. japonica* when under the chilling condition. Additionally, most of the PFP and CP related genes being identified to be upregulated, and the accumulated content of CGA, luteoloside, anthocyanins, and carotenoids indicated the PFP and CP were significantly activated by the chilling stress. The results above indicate that Ca^2+^ signaling might promote the biosynthesis of flavonoids and carotenoids in the chilling treated leaves of *L. japonica* through inducing the accumulation of BR and activity the BR signaling regulators.

## 5 Conclusions

**Figure 11:**
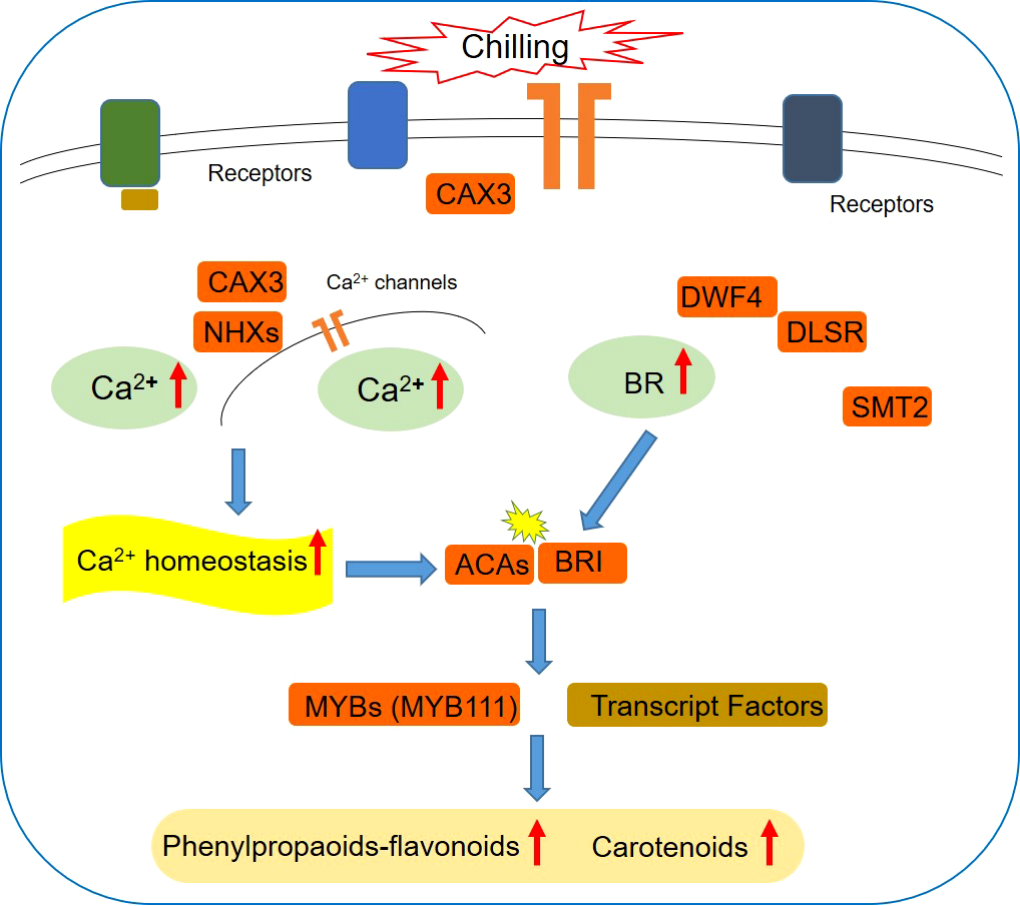
The overview of major findings in this study.

In this study, comparative transcriptomics was carried out to reveal the molecular mechanism underlying the physiological phenotype of *L. japonica* under chilling stress. The results are as follows: (1) the leaves color was changed from green to purple and the content of carotenoids and anthocyanins were increased under chilling stress; (2) DEGs were mainly enriched in secondary metabolism, lipids, cell wall, and minor carbohydrate; (3) the UGs were functionally categorized in protein metabolism, RNA, transport, signaling, and cell metabolism; (4) the interacted DEGs in transport and signaling processes were functioned in transition ion transport and calcium transport/potassium ion transport; (5) *NCL*, *NHX2*, *NCRK*, *ACA9*, *CAX3*, *ACA8* involved in the regulation of calcium homoeostasis were upregulated and (6) BRI, *DWF4*, *DLSR*, and *SMT2* involved in BR biosynthesis and signaling were all upregulated in response to chilling stress; (7) the accumulation of CGA and luteoloside was increased in leaves of *L. japonica* under chilling stress. The results guide to form the overview of response mechanism of *L. japonica* to chilling stress (**Figure 11**): the calcium concentration was increased and the calcium homeostasis was regulated to a high level under the effect of signaling and transporter related genes, thereby triggering BR signal activity, by which the biosynthesis of flavonoids and carotenoids were promoted through the induction of related transcription factors. The changed ratios of pigments and accumulation of CGA and luteoloside indicate that the experimental chilling condition could be developed to artificial strategy to promote both the medicinal quality and horticultural value of *L. japonica*.

## Data availability statement

The data presented in the study are deposited in the National Center for Biotechnology Information Sequence Read Archive repository, accession number PRJNA903538.

## Author contributions

BY and LZ conceived and designed the study. MZ wrote the manuscript. ML performed the bioinfomatic analysis. MZ, KW and XT performed the material treatment and collection, library construction, and qRT-PCR. RQ performed the pigment content measurement. JL, SG, and HF performed the HPLC analysis of metabolites. ZZ did the data integration and function annotation. BY and LZ reviewed and edited the manuscript. All authors contributed to the article and approved the submitted version.
