# Supplementary material for: Transcriptomic analysis unravels the molecular response of Lonicera japonica leaves to chilling stress
Source: Front Plant Sci. 2022 Dec 22;13:1092857. doi: 10.3389/fpls.2022.1092857 (PMC9815118; doi:10.3389/fpls.2022.1092857)
Supplement: Supplementary file 1 [file Table_1.docx]

Supplemental Table 1. Primer sequences used for qRT-PCR

| Gene | Sense Primer | Anti-sense Primer |
| --- | --- | --- |
| NRAMP3 | GTGCTTCAGTCGGTTCAGATAC | CACCAACGCAGCCACAATC |
| ABCG1 | CTTCTTCAGCATCTTCGTCTCA | CGATTGTGTAGCCAAGCATTAC |
| ZAT | TTGCCATCTCCTTGTTCTCTT | AGCCATTGACCTTACCTGTATC |
| ABCI12 | GCGTGTTGGATTACTCGTGTT | ACATTGAAGGCGGTGGTGAT |
| AT3G21690 | AGCGTCGGTCGTTCTAACT | GCCACAGATTCAGATTCACCTA |
| ABCG37 | ATATCACCACCGAGCGAACT | CGAAGCCGACCAGTAATACC |
| MTP8 | GAGATTGTCCGTGCCTATGC | TCCAGAACAGTTCCAGACCAA |
| ABCC14 | CCGTCATCCATCACTTCTCAGA | AAGCCAAGCCACTCATTCAATC |
| ABCI10 | CACTAAGCGGCGGTCAGAA | CTCTTCCAAGCGGTGAGTTAC |
| HMA1 | CCAAGCCTGCTGTCAAGTCT | GCCGCAAGAGCCATCAGTA |
| MFL1 | TCGGCTACATTAACAACTCCTT | ACCCAACCTTCATCCCTCAA |
| YSL3 | GCTCAATCTTACCACTGGACTT | TATAGCACGCAACAGCACAT |
| NCL | TGCTCTTCCTGATGCTATGCT | CACTTGCCAACCACAACACA |
| NCRK | TGATGGTAGAATTGTGGCAGTT | CAGAGCAGTATCCGATTAAGGT |
| NHX2 | ACTATCTACTTCCGACCACACT | ATCCACCGATTCTCCTCCAATA |
| ACA9 | TTAATGTTGTGGCGGCAGTT | TTGTCAGTTGGTGGTTCAGTAG |
| CAX3 | GATGGTGATGATGTGGCTGAG | CCAAGATTCTGATGCGGACTC |
| RAC6 | TCTTATACCAGCAACACCTTCC | CTCCCATTGACGACCACATTT |
| CPLEPA | AACTACTGCTCCAAGTGTTGTT | CCAGCTTCAGGAAGTAAGGATG |
| ABCB25 | GATGATGCGAAGCCTCTGAC | TACCACTGCCACTTGTTCCA |
| RABF2A | GCTGGTGCTTCTTGGAGAC | TTGTCGGTTCGTGGTAATCG |
| EMB269 | CGGAGATGTGGTTGGAGTTAGA | GAGCAGCACTGATGACACTAAG |
| ACTIN | TGCGACAATGGAACTGGAATG | GCCGACATAAGCATCCTTCTG |
