# Supplementary material for: Transcriptomic analysis unravels the molecular response of Lonicera japonica leaves to chilling stress
Source: Front Plant Sci. 2022 Dec 22;13:1092857. doi: 10.3389/fpls.2022.1092857 (PMC9815118; doi:10.3389/fpls.2022.1092857)
Supplement: Supplementary file 3 [file Presentation_1.pptx]

## Slide 1
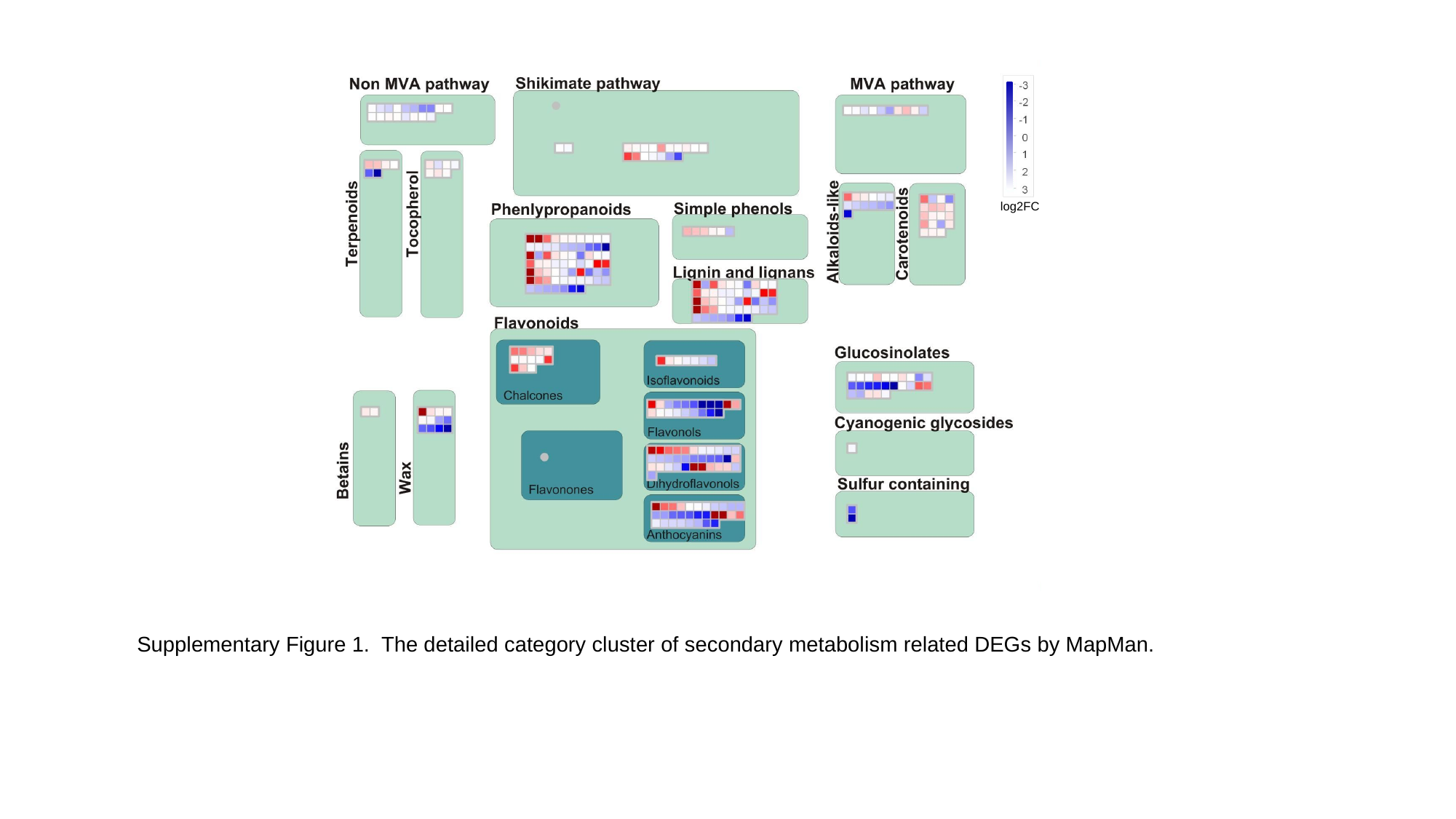

log2FC
Supplementary Figure 1. The detailed category cluster of secondary metabolism related DEGs by MapMan.

## Slide 2
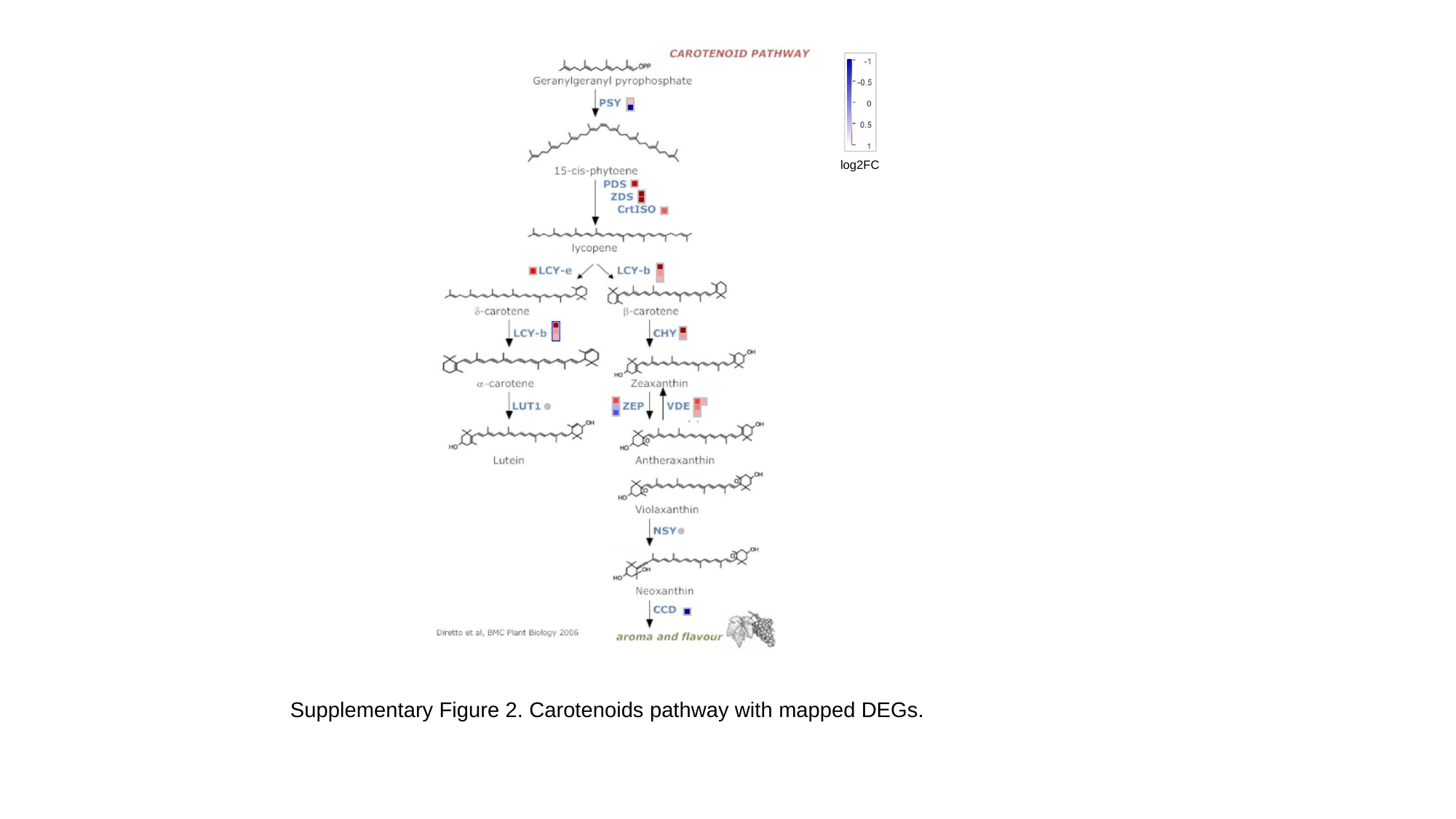

log2FC
Supplementary Figure 2. Carotenoids pathway with mapped DEGs.

## Slide 3
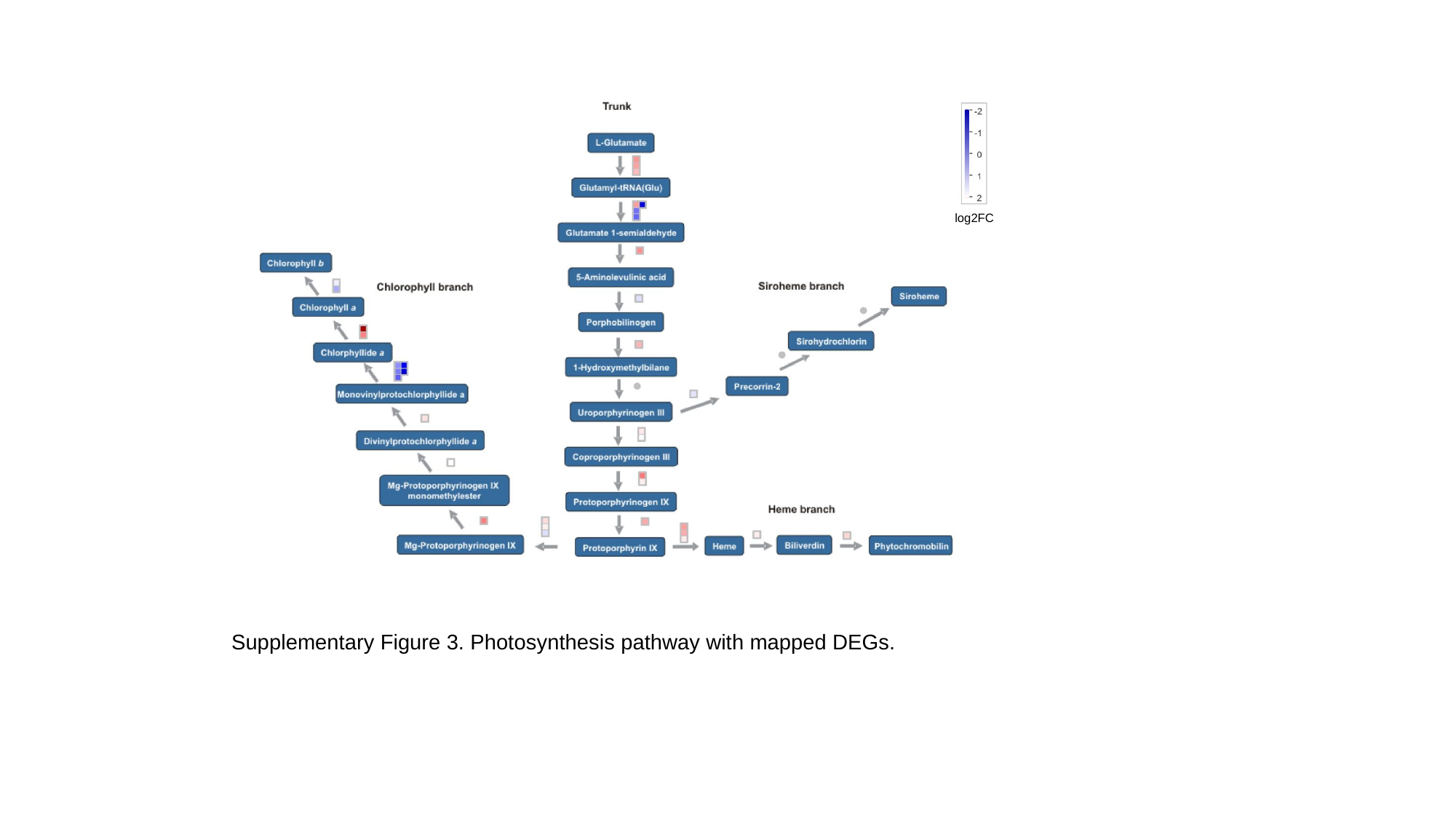

log2FC
Supplementary Figure 3. Photosynthesis pathway with mapped DEGs.

## Slide 4
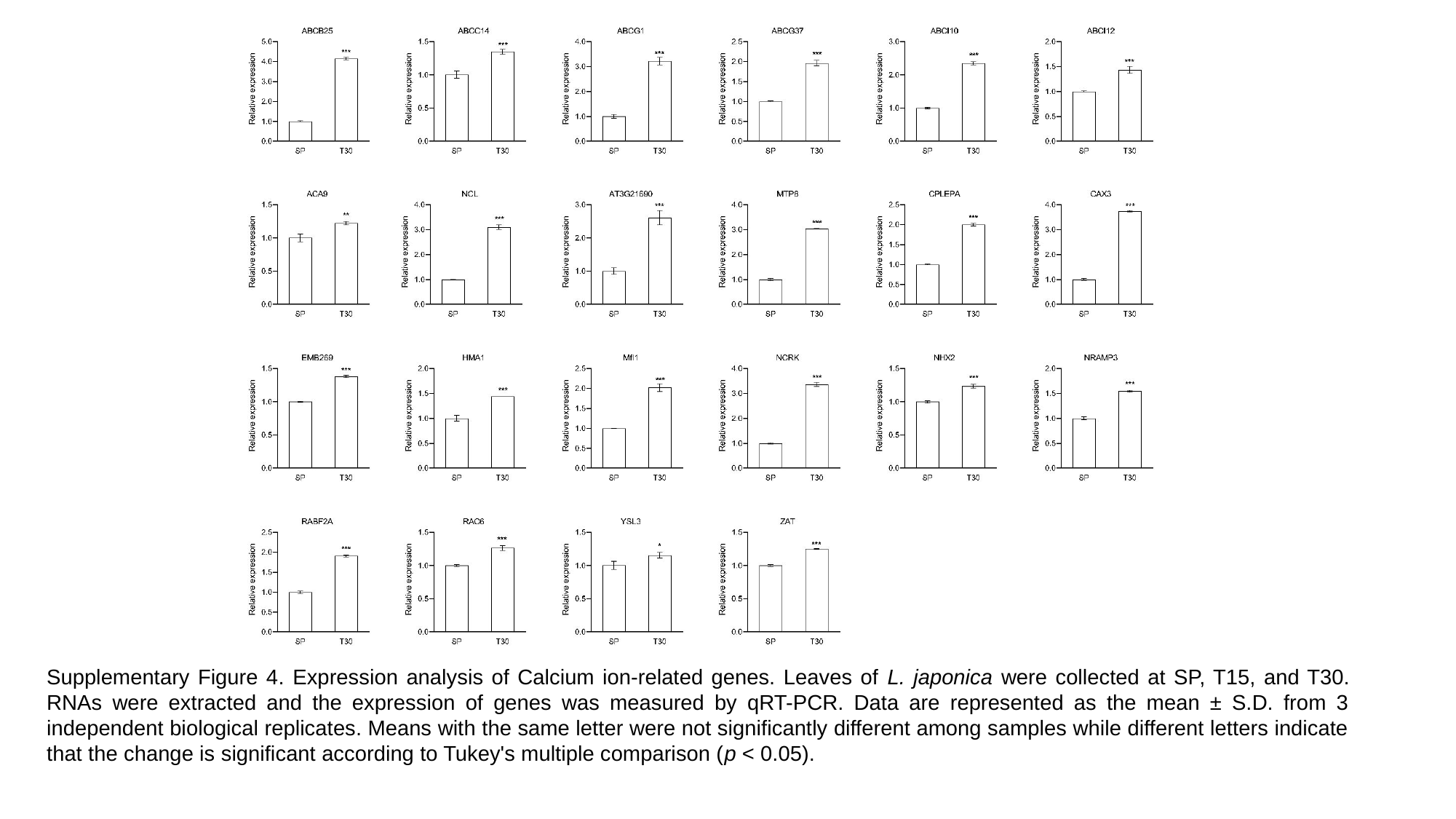

Supplementary Figure 4. Expression analysis of Calcium ion-related genes. Leaves of L. japonica were collected at SP, T15, and T30. RNAs were extracted and the expression of genes was measured by qRT-PCR. Data are represented as the mean ± S.D. from 3 independent biological replicates. Means with the same letter were not significantly different among samples while different letters indicate that the change is significant according to Tukey's multiple comparison (p < 0.05).
